# Environmental Barcoding: A Next-Generation Sequencing Approach for Biomonitoring Applications Using River Benthos

**DOI:** 10.1371/journal.pone.0017497

**Published:** 2011-04-13

**Authors:** Mehrdad Hajibabaei, Shadi Shokralla, Xin Zhou, Gregory A. C. Singer, Donald J. Baird

**Affiliations:** 1 Biodiversity Institute of Ontario, Department of Integrative Biology, University of Guelph, Guelph, Ontario, Canada; 2 Environment Canada, Canadian Rivers Institute, Department of Biology, University of New Brunswick, Fredericton, New Brunswick, Canada; King Abdullah University of Science and Technology, Saudi Arabia

## Abstract

Timely and accurate biodiversity analysis poses an ongoing challenge for the success of biomonitoring programs. Morphology-based identification of bioindicator taxa is time consuming, and rarely supports species-level resolution especially for immature life stages. Much work has been done in the past decade to develop alternative approaches for biodiversity analysis using DNA sequence-based approaches such as molecular phylogenetics and DNA barcoding. On-going assembly of DNA barcode reference libraries will provide the basis for a DNA-based identification system. The use of recently introduced next-generation sequencing (NGS) approaches in biodiversity science has the potential to further extend the application of DNA information for routine biomonitoring applications to an unprecedented scale. Here we demonstrate the feasibility of using 454 massively parallel pyrosequencing for species-level analysis of freshwater benthic macroinvertebrate taxa commonly used for biomonitoring. We designed our experiments in order to directly compare morphology-based, Sanger sequencing DNA barcoding, and next-generation environmental barcoding approaches. Our results show the ability of 454 pyrosequencing of mini-barcodes to accurately identify all species with more than 1% abundance in the pooled mixture. Although the approach failed to identify 6 rare species in the mixture, the presence of sequences from 9 species that were not represented by individuals in the mixture provides evidence that DNA based analysis may yet provide a valuable approach in finding rare species in bulk environmental samples. We further demonstrate the application of the environmental barcoding approach by comparing benthic macroinvertebrates from an urban region to those obtained from a conservation area. Although considerable effort will be required to robustly optimize NGS tools to identify species from bulk environmental samples, our results indicate the potential of an environmental barcoding approach for biomonitoring programs.

## Introduction

Understanding biodiversity is fundamental to ecological research and key to maintaining a healthy environment and a sustainable economy. However, biodiversity science remains the study of unknowns. Over 1.9 M species have been formally described since Linnaeus first started the task 250 years ago, yet it is estimated that 10–100 M species exist on Earth [1], [2]. Therefore, not only is our characterization of biodiversity painstakingly slow, but the fact that there is order-of-magnitude uncertainty in our best estimate for the totality of Earth's biodiversity [2] suggests that current tools and techniques are inadequate for the task of accurate assessment. “What is the species composition of a particular ecosystem?” “How does biodiversity change over time, space, and in relation to future environmental change?” are both fundamental questions we try to answer through biomonitoring programs, by employing biotic surveys to assess change in threatened habitats. Both questions are difficult to answer in a consistent and timely fashion, and nearly impossible to implement as monitoring objectives. As a consequence of the sensitivity of species to pollution and other disturbances which alter their habitat, environmental agencies are increasingly choosing biomonitoring approaches to assess ecosystem status and trends. However, accurate (i.e. avoiding mis-identification) and consistent (level of taxonomic identification e.g. family/genus/species) taxon identification has proved difficult to achieve using traditional morphological approaches. This is particularly true for the large-scale application of macroinvertebrate sampling in river biomonitoring, where larval stages are often difficult or impossible to identify below the level of taxonomic family. This issue has caused difficulties in implementing large-scale biomonitoring programs, particularly in relatively less-populated countries such as Canada, where remoteness poses a significant logistic challenge for sample collection, coupled with poor knowledge of the local fauna.

Sanger's invention of DNA sequencing revolutionized all branches of the biological sciences [3]. In biosystematics, DNA sequence information provides vast amounts of reproducible and robust genetic data that can be informative at nearly any level of taxonomic hierarchy: from individuals in populations, to species, to the deepest branches of the Tree of Life. DNA sequence-based analyses have provided evolutionary biologists and ecologists the opportunity to address questions they could not answer using other types of data. In recent years—particularly with the introduction of the concept of DNA barcoding in 2003 [4]—efforts have been directed towards building a standard sequence library for all eukaryotes by focusing DNA sequencing efforts on small, species-specific portions of the genome called DNA barcodes [5], [6]. The primary utility of DNA barcoding is to identify unknown specimens at the species-level by comparing the query sequence to a DNA barcode reference library built based on known species [7]. In addition, patterns of sequence variation can be used to flag new and cryptic species. By sampling more genes or individuals, DNA barcode projects can shift to population-level analysis or deep phylogenetic questions [7]. In the past seven years, over 1.1 M individuals from about 95,000 species have been added to the DNA barcode library [8]. This number is not significant in the context of the 1.9 M known and 10–100 M estimated unknown species [1], [2]. However, this progress is significant because DNA barcoding in the past seven years has chiefly been geared towards proof-of-concept projects to enhance application through the development of improved protocols [9], [10], [11]. Major hurdles in the high-throughput analysis of DNA barcodes have been resolved and single analytical facilities can now process several hundred thousand samples per year [10]. Global projects such as the International Barcode of Life project (iBOL, http://www.ibolproject.org/) and other concerted efforts to barcode taxonomic groups or regional biota will rapidly increase the sequence coverage in DNA barcode libraries.

Although Sanger-based DNA sequencing has proved robust for building large sequence libraries such as DNA barcode reference libraries, it is not a feasible approach for tackling bulk environmental samples because these samples can contain thousands of individuals from hundreds of species ranging from bacteria to higher eukaryotes. Separating these individuals and then using single-specimen Sanger sequencing has historically been challenging and for some material is beyond the scope of traditional technologies. Although cloning followed by sequencing a library of cloned fragments partially addresses this problem, this method has its own limitations and can introduce biases. Consequently, biomonitoring programs and other large-scale biodiversity analyses in ecological and environmental studies cannot be performed routinely on a large-scale using a single-specimen Sanger sequencing workflow. In other words, although it is possible to use 96-well and even larger assemblages of specimens in conventional Sanger sequencing, it is cumbersome to separate and sort each individual organism into sets of 96 samples for processing. A typical environmental sample includes hundreds to thousands of organisms and a biomonitoring regime often requires multiple environmental samples that are repeated over time and space. Hence, the bottleneck in this case may not only be at the DNA sequencing step but can also occur at the collection, sorting, and preparation steps. Working with specimens in a one-at-a-time fashion, is tedious, time-consuming, and expensive.

Soon after the introduction of so-called “next-generation” DNA sequencers in 2005 [12], biodiversity analyses became an important application for these technologies [13]. Since longer sequence length means better taxonomic resolution, the 454 Genome Sequencer FLX is the preferred NGS platform for biodiversity studies [12] as it is capable of providing 250–400 base long sequence reads versus less than 100 bases for the two competing platforms. This property is important because DNA fragments (e.g. PCR products) that are sequenced in each sequencing reaction will be examined bioinformatically to derive biodiversity measures from a given environmental samples. It has been shown that longer sequences can provide more accurate biodiversity information such as species-level resolution [14]. The majority of biodiversity studies using this equipment have targeted prokaryotic biodiversity in different environmental samples, from the ocean floor [13] to human micro-flora [15]. These studies typically use sequence variation in a short fragment of ribosomal genes (e.g. 16S rDNA) for estimating the diversity of bacteria in the sample. The results are compared to a relatively large sequence library of 16S genes using statistical clustering methods such as BLAST [16]. The same approach can be applied to large environmental samples of eukaryotic organisms. It has been shown that a small mini-barcode fragment of the mitochondrial cytochrome c oxidase 1 (COI) DNA barcode sequences—a sequence length that can readily and robustly be obtained through 454 pyrosequencing—can provide the information required for identification of individual species with more than 90% species resolution [11], [14], [17].

**Figure 1:**
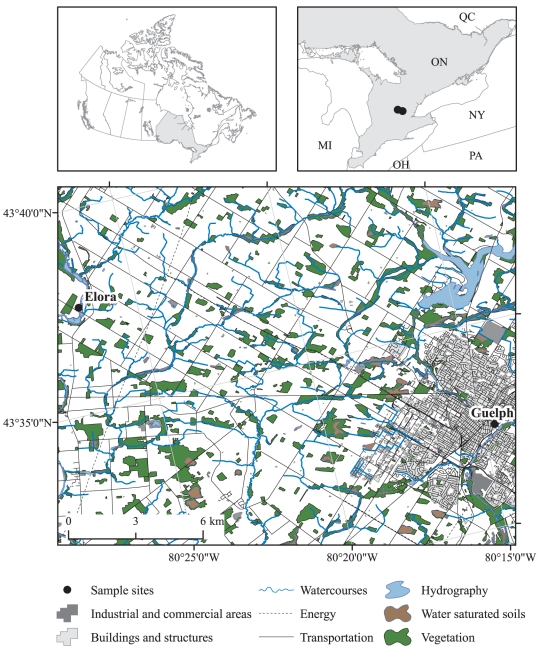
A map of sampling locations in an urban setting (Speed River, Guelph, Ontario) and near a conservation area (Grand River, Elora, Ontario).

Since early 2008, we have started a technology development project to utilize NGS in biomonitoring programs. We established a NGS facility at the Biodiversity Institute of Ontario, aimed at reconstructing the species composition of environmental samples of eukaryotes. Here we present our preliminary work on samples collected at two locations (Figure 1) focused on two of the more important freshwater macroinvertebrate groups: caddisflies (Trichoptera) and mayflies (Ephemeroptera).

## Results

### Environmental barcoding of pooled known mixtures

**Figure 2:**
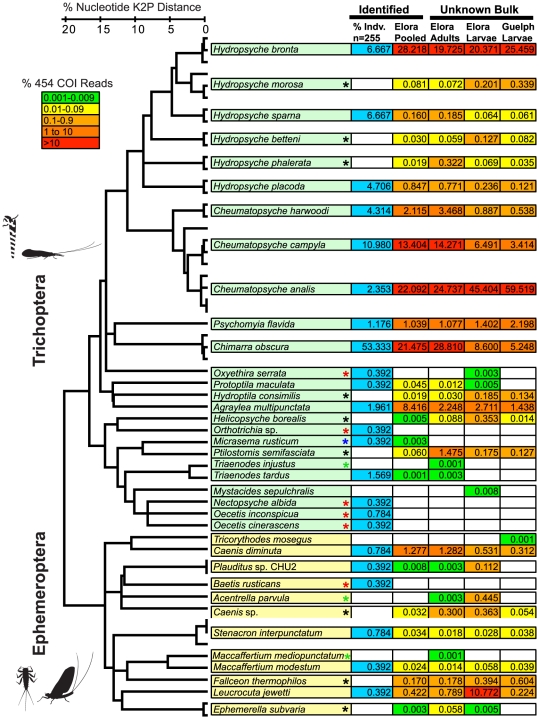
Species composition of mayflies (Ephemeroptera) and caddisflies (Trichoptera) from three bulk samples and a pooled sub-sample, obtained through 454 pyrosequencing of a 130 base COI mini-barcode. Species composition based on Sanger sequencing barcodes of a subsample of 255 individuals is shown in the blue column. A neighbor-joining tree diagram based on K2P model of nucleotide substitution of COI mini-barcodes for all haplotype sequences demonstrates species-level resolution. Pattern of species diversity in each sample is shown by color-coded rectangles based on percentage of pyrosequence reads for each species. Color asterisks represent species absent in specific environmental samples.

Our first experiment involved standard COI barcode analysis from 255 specimens (a single leg from each adult insect) from a single benthic sampling event in the Grand River at Elora, Ontario (Figure 1). This analysis revealed the presence of 23 species of Ephemeroptera and Trichoptera (Figure 2). These species were compared to a larger library of Ephemeroptera and Trichoptera haplotypes previously obtained at the study sites (Figure 2, tree diagram). Interestingly, as illustrated in Figure 2, a 130-base mini-barcode fragment of COI was suitable to separate species (and their haplotypes) in the Ephemeroptera and Trichoptera taxa studied here. In our next experiment we pooled the whole bodies from these 255 specimens, extracted DNA from this slurry, amplified 130-base COI mini-barcodes and sequenced the amplicons using a 454 pyrosequencer. This analysis generated sequences from 17 of the 23 species that were originally pooled, including all species that were represented by at least 1% of the individuals (Figure 2, Elora Pooled). The 6 missing species were all uncommon, each represented by only 1 or 2 individuals in the mixture (Figure 2, red asterisks). Surprisingly, sequence records were also detected for 9 species of Trichoptera and Ephemeroptera known to occur in the area but that were not in the pooled mixture (Figure 2, black asterisks). In total, we recovered barcode sequence signatures of 26 species in the pooled adult mixture (Figure 2, Elora Pooled).

### Environmental barcoding of unknown bulk specimens

The next set of experiments focused on comparing the species compositions of unknown bulk environmental samples collected from the urban site (Speed River, Guelph) and the conservation area site (Grand River, Elora) using 454 pyrosequencing approach. We first focused on a bulk sample of adults from the Grand River in Elora as a direct comparison with our earlier experiment involving pooled samples. The analysis of pooled identified specimens by pyrosequencing had revealed 26 distinct species (Figure 2, Elora Pooled). Direct 454 pyrosequencing of the bulk adults sample found 28 distinct species, missing one from the pooled analysis (blue asterisk in Figure 2) but including three that were missed by the pooled analysis (green asterisks). Our final analysis involved a comparison of larval communities in the Speed and Grand Rivers (Guelph and Elora, respectively). This experiment was done based on a sampling approach commonly used by environmental agencies for biomonitoring applications. Our environmental barcoding analysis revealed the presence of 22 and 27 species in Speed and Grand Rivers, respectively. The species composition of the larval sample from the Grand River (Elora) corresponded with the adult assemblage from the same location although 5 species with low percentage of pyrosequence reads were not common to the two samples (Figure 2).

## Discussion

Next-generation sequencing is increasingly being used in metagenomics studies to determine the occurrence of microbial taxa. For small-sized taxa which are difficult to culture, next-generation sequencing technologies have proved useful in revealing their biodiversity, or for the comparative analysis of microbial biota [13], [15]. However, aside from a few studies—mainly focused on data analysis and sequencing error rates—next-generation sequencing has not been directly compared to other identification methods especially for eukaryotic biota. Here we designed and executed our experiments to make comparisons between 454 pyrosequencing and traditional Sanger sequencing based DNA barcoding. Our aim has been to evaluate the feasibility of 454 pyrosequencing to overcome two important challenges faced by biodiversity researchers and environmental agencies using benthic macroinvertebrates for their studies. The first challenge is sorting and analysing small specimens especially larvae that are typically used in benthic biodiversity analysis–one-by-one. This issue is both technically challenging and very labour-intensive and is therefore a bottleneck in morphological identifications as well as Sanger sequencing based DNA barcode analysis (see above). The second problem, which is mainly encountered in morphological analysis, is species-level identification, which is often impossible to achieve in larval samples. Our results show the effectiveness of 454 pyrosequencing for the analysis of an engineered mixture of adult insects. We were able to gain species-level resolution for all abundant species in the mixture (Figure 2). However, 454 pyrosequencing missed 6 low abundance species but provided DNA sequence evidence for the presence of 9 other species undetected in single specimen Sanger-based analysis. What might appear to be a puzzling result can be explained as a consequence of two issues. The first issue, which can explain failure in identification of 6 lower abundance species, is the bias associated with binding PCR primers to target-template DNA in a mixture. Species with higher affinity in their primer binding sites and/or species with higher abundance (i.e. more biomass in a bulk sample) can capture more primer molecules during the process of PCR annealing. Consequently, species with lower affinity to primers and/or lower abundance (i.e. less biomass in a bulk sample) may not yield amplicons. Deeper next-generation sequencing can potentially alleviate this issue. Other studies, especially studies targeting rare HIV mutants have used this strategy to detect low abundance virus genes in clinical samples [18]. The second issue, which can explain the detection of DNA sequences from 9 species that were not originally present in the specimens we pooled in this analysis, is likely the result of carryover of DNA from these 9 species from the liquid preservation media (in this case Ethanol) to the bodies of specimens selected for the pooling experiment. The authors have recently shown that DNA from specimens can be detected directly from preservative ethanol [19]. In addition, we have been able to obtain DNA sequences from majority of species in a mixture by directly 454 pyrosequencing the ethanol used as preservative for bulk benthic samples (results not shown). Because the total number of species detected from a single 454 pyrosequencing analysis is larger when compared to single specimen Sanger-based DNA barcoding, and yet all common species are detected in 454 analysis, we believe 454 pyrosequencing is advantageous as compared to a single specimen approach.

Our work for the first time used the standard COI DNA barcode information in 454 pyrosequencing approach for the analysis of specimens from two orders of insects. Although the majority of prior studies that employed 454 pyrosequencing for biodiversity surveys have focused on ribosomal markers such as 16S rDNA (in bacteria) and 18S rDNA (in protists and meiofauna) we decided to use COI firstly because a small mini-barcode sequence of this gene allows species-level resolution in most animal and protist groups tested [14] and second, it can be linked to an expanding DNA barcode reference library [8]. Lack of universal primers has been used as an argument against the use of this gene region in next-generation sequencing analysis of environmental samples [20]. However, our results show that COI PCR primers can be effective in amplifying multiple templates. The bias associated to COI is comparable to reported bias associated with other genes [20]. This bias can obscure quantitative analysis of species abundance and can also negatively influence the detection of low abundance species when sequencing depth is not maximized. Nevertheless, our analysis shows that the percentage of sequence reads obtained in environmental barcoding using 454 pyrosequencing is comparable to the abundance measure obtained through counting individuals in the bulk sample (Figure 2). This is perhaps due to the fact that the relative abundance of species in nature (i.e. their number in a bulk sample) can potentially offset any bias from primer-binding in PCR. In addition, species-level resolution gained in COI analysis, coupled with linkages to standard DNA barcode libraries can be advantageous as compared to other less variable markers, that may not provide species-level resolution (i.e. 18S rDNA).

The ability to automate a biodiversity survey of, for example, bulk macroinvertebrate samples can revolutionize large-scale biomonitoring programs that are costly, labour-intensive and time-consuming to implement across large geographic regions. Moreover, the ability to cheaply and rapidly sequence material from different habitats not only increases the efficiency of biomonitoring as a technique, but it expands the scope of monitoring programs, by extension into habitats and biota groups which are currently not studied due to poor taxonomic knowledge or technical competency. Our 454 pyrosequencing analysis of bulk larval samples collected in two contrasting sampling locations shows promise for direct and immediate application in routine biomonitoring studies. Seven species were not common to both Speed (2 unique) and Grand River (5 unique) larval samples although they were collected on the same day (Figure 2). Moreover, Guelph larval samples represented 5 fewer species in total as compared to larval samples from Elora. The results obtained indicate clear differences in faunal composition, even within this restricted set of organisms–such differences are typically due to habitat variation between sites (e.g. river flow conditions, thermal regime) but may also be due to direct anthropogenic influence (e.g. chemicals in municipal wastewater effluent, sedimentation from construction activities in the riparian zone). These observations, if expanded to include additional samples, could be used to indicate differences in the ecological quality of urban versus conservation habitat. Here, we have presented a pilot study: in future, studies involving a more comprehensive sampling across time and space to compare conventional biomonitoring results to DNA-based biodiversity analysis are urgently required to evaluate the feasibility of our approach. In addition, there is a clear need for data analysis algorithms and specialized bioinformatics and visualization tools to facilitate rapid, robust, and repeatable interpretation of sequencing results [21], [22]. This is especially important because monitoring applications require repeated sampling and timely analysis, both requiring reliable computational tools. Future advancements in environmental barcoding will make biomonitoring faster, cheaper, and more accessible to regulatory agencies, industry and the research community.

## Materials and Methods

### Sampling strategy

Two sampling locations from two nearby rivers from the same watershed were selected for this study. The first sampling location is in the Speed River in an urbanized region (Guelph city, Ontario, Canada) and the second is in the Grand River near a conservation area (Elora, Ontario, Canada). The distance between the two sites is approximately 22 km (Figure 1). Both adult and larval samples were obtained from the two sites during summer 2008. The adult samples were collected in 95% ethanol using a light trap while the larval samples were collected using the Environment Canada's standard benthic macroinvertebrate collection method, a three minute travelling kick-net covering a variety of aquatic habitat types.

### Experimental design and DNA barcode analysis

From the adults bulk sample collected in Elora, we sampled 255 individuals representing 23 different species of Ephemeroptera and Trichoptera. These specimens were morphologically identified and sorted in 96-well plates. A single leg from each individual was then subjected to routine DNA barcoding following standard COI DNA barcoding protocols [10]. We amplified standard full-length (650 bp) COI DNA barcodes in two PCR amplifications using LepF1/LepR1 [23] and LCO1490_tl/HCO2198_tl primers [24] using a standard pre-made PCR mixture followed by standard Sanger sequencing in an ABI 3730XL DNA sequencer. Details of DNA barcodes obtained by Sanger sequencing are available on the Barcode of Life Data System (BOLD) website [8]. The rest of the bodies were homogenized using MP FastPrep-24 Instrument (MP Biomedicals Inc.) and its DNA was extracted from the slurry (see below for details on DNA extraction). The rest of the adults and benthic larval samples were processed as bulk specimens using a similar homogenization step.

### DNA extraction and PCR optimization from bulk environmental samples

Bulk samples were mixed in a conventional shaker and the resultant slurry was incubated at 56°C for approximately two hours to evaporate residual ethanol. For each sample, we divided one gram in 10 MP lysing matrix tubes “A” (100 mg each) and homogenized them using an MP FastPrep-24 Instrument (MP Biomedicals Inc.) at speed 6 for 40 sec. Total DNA of this homogenized slurry (both for adults and larval samples) was extracted using Nucleospin tissue kit (Macherey-Nagel Inc.) following manufacturer's instructions and eluted in 70 µl of molecular biology grade water. We combined 10 independently isolated DNA extracts for each sample in one tube (700 µl total volume). The COI minibarcode (130 bp) was amplified using a commonly used forward primer LepF1: 5′-ATTCAACCAATCATAAAGATATTGG-3′ [23] and a newly designed reverse primer, EPT-long-univR: 5′-AARAAAATYATAAYAAAIGCGTGIAIIGT-3′ in a two-step PCR amplification regime. The first PCR used COI specific primers and the 2^nd^ PCR involved hybrid 454 fusion-tailed primes. In the first PCR ten amplicons were generated for each environmental sample. Each PCR reaction contained 2 µl DNA template, 17.5 µl molecular biology grade water, 2.5 µl 10× reaction buffer, 1 µl 50× MgCl_2_ (50 mM), 0.5 µl dNTPs mix (10 mM), 0.5 µl forward primer (10 mM), 0.5 µl reverse primer (10 mM), and 0.5 µl Invitrogen's Platinum Taq polymerase (5 U/µl) in a total volume of 25 µl. The PCR conditions were initiated with heated lid at 95°C for 5 min, followed by a total of 15 cycles of 94°C for 40 sec, 43.5°C for 1 min, and 72°C for 30 sec, and a final extension at 72°C for 5 min, and hold at 4°C. PCR success was checked by Agarose gel electrophoresis. Amplicons from each environmental sample were pooled and subjected to purification using Qiagen's MiniElute PCR purification columns and eluted in 50 µl molecular biology grade water. The purified amplicons from first PCR were used as templates in a second PCR (10 reactions per environmental sample) with similar conditions as the first PCR with the exception of using 454 fusion-tailed primers in a 30-cycle amplification regime. The second PCR was done to attach fusion tails to allow subsequent 454 emulsion PCR. We used an Eppendorf Mastercycler ep gradient S thermalcycler in all PCRs. A negative control reaction (no DNA template) was included in all experiments.

### 454 Pyrosequencing

All amplicons were sequenced on a 454 Genome Sequencer FLX System (Roche Diagnostics GmbH) following the amplicon sequencing protocol. Amplicons of each sample was bi-directionally sequenced in 1/4^th^ of full sequencing run (70×75 picotiter plate) with the exception of our 255 pooled adults that was sequenced in 1/8^th^. This sample was subsequently sequenced in 1/4^th^ run with same results as 1/8^th^ run (data not shown). The total number of sequence reads for each sample was as follows: 255 pooled adults from Elora 38,147; bulk adults from Elora 79,081; bulk larvae from Elora 90,495; bulk larvae from Guelph 78,213. All sequence data have been deposited into GenBank (accession numbers SRA029661.2, SRA029662.1, SRA029663.1, SRA029664.1, SRA029665.1, SRA029666.1, SRA029667.1, SRA029668.1). Details of the 454 pyrosequencing run are available by request from the corresponding author. Pyrosequencing reads were compared against a reference Sanger library of COI sequences of Trichoptera and Ephemeroptera from the BOLD database [8], using NCBI's Megablast program. Reads that had a unique best-hit with an identity score greater than 98% were considered to be positive matches. A neighbour-joining tree with K2P distances from all haplotypes from species found in pyrosequencing analysis was constructed using Mega 4.1 [25].
